# Neuroplastic Effect of Exercise Through Astrocytes Activation and Cellular Crosstalk

**DOI:** 10.14336/AD.2021.0325

**Published:** 2021-10-01

**Authors:** Fengwu Li, Xiaokun Geng, Ho Jun Yun, Yazeed Haddad, Yuhua Chen, Yuchuan Ding

**Affiliations:** ^1^China-America Institute of Neuroscience, Luhe Hospital, Capital Medical University, Beijing, China.; ^2^Department of Developmental Cell Biology, Key Laboratory of Cell Biology, Ministry of Public Health, and Key Laboratory of Medical Cell Biology, Ministry of Education, China Medical University, Shenyang, China; ^3^Department of Neurology, Beijing Luhe Hospital, Capital Medical University, China.; ^4^Department of Neurosurgery, Wayne State University School of Medicine, Detroit, MI, USA.

**Keywords:** rehabilitation, neuroplasticity, neuron, microglia, interaction

## Abstract

Physical exercise is an effective therapy for neurorehabilitation. Exercise has been shown to induce remodeling and proliferation of astrocyte. Astrocytes potentially affect the recruitment and function of neurons; they could intensify responses of neurons and bring more neurons for the process of neuroplasticity. Interactions between astrocytes, microglia and neurons modulate neuroplasticity and, subsequently, neural circuit function. These cellular interactions promote the number and function of synapses, neurogenesis, and cerebrovascular remodeling. However, the roles and crosstalk of astrocytes with neurons and microglia and any subsequent neuroplastic effects have not been studied extensively in exercise-induced settings. This article discusses the impact of physical exercise on astrocyte proliferation and highlights the interplay between astrocytes, microglia and neurons. The crosstalk between these cells may enhance neuroplasticity, leading to the neuroplastic effects of exercise.

## 1. Introduction

Neuroplasticity is a process characterized by enhanced neurogenesis, synaptogenesis, angiogenesis and release of various neurotrophic factors [1]. The brain changes its functional and structural properties of neuroplasticity, which commonly occurs during learning or acquiring new skills. Evidence suggests that physical exercise facilitates neuroplasticity in certain areas of the brain, promoting cognitive and motor functions [2, 3]. Studies have shown potential mechanisms by which exercise could influence neuroplasticity, including neurogenesis, growth factor production, alteration of neuronal excitability and axonal outgrowth [4-6]. For instance, astrocytes exhibit plasticity via changes in their morphology and functional modification in response to stimuli [7]. They play a critical role in recovery from nerve injuries by interacting with blood vessels and microglia [8]. Numerous evidences highlights the roles of astrocytes in neural circuits of developing long-term adaptations under various physiological and pathological conditions [9, 10].

Despite astrocytes being shown to be activated after physical exercise [11], their roles or interactions with neurons and microglia, and any subsequent neuroplasticity have not been extensively studied in exercise-induced settings [11]. Without knowing the mechanisms of the exercise-induced neuroplasticity, it still remains challenging to establish optimal exercise programs for rehabilitation [4]. This article reviews the related studies, and provides descriptions outlining the role of astrocytes in exercise settings. Particularly, the interplay between astrocyte, microglia and neuron is emphasized. Understanding the roles of astrocytes in physical exercise and the effects on neuroplasticity could provide opportunities to improve long-term recovery from central nervous system (CNS) injuries.

## 2. Physical Exercise Enhances Neuroplasticity

Exercise therapy has long been considered a reliable strategy to ameliorate physical disabilities by inducing neuroplasticity [12, 13]. Physical exercise is beneficial for those with neurodegenerative disorders [14] and neurovascular injuries [15], because it improves their behavioral function by promoting blood flow and neurogenesis [16]. Exercise increases the rate of newborn cell numbers, the fraction differentiating into neurons, and the proportion that incorporates into neuronal circuits; these can be beneficial for functional motor outcomes [16]. Physical exercise - particularly balance and coordination movements - facilitates synaptic plasticity and angiogenesis in the brain [17, 18]. Involuntary, voluntary, and forced exercises have been found to induce high expressions of synaptic plasticity molecules, such as postsynaptic density 95 (PSD-95), synapsin I (SYN), and angiogenesis proteins, such as vascular endothelial cell growth factor (VEGF). This upregulation in the hippocampus or cortex surrounding ischemic regions ultimately results in enhanced cognition and better functional rehabilitation [19].

Synaptic plasticity is the capacity of neurons to undergo activity-dependent changes in their intensity and efficacy of synaptic transmission. This process, presumably the major mechanism of neuroplasticity, is responsible for learning and memory as well as development and response of the brain to injuries [20]. Studies involving animals with stroke show increased brain-derived neurotrophic factor (BDNF), insulin-like growth factor-I (IGF-I) and nerve growth factor (NGF) after physical exercise [17, 21, 22]; these factors all contribute to reduction of neurons loss and growth of synaptic connections in multiple brain regions [11]. Physical exercise regulates numerous supporting systems of neuroplasticity, including neurogenesis, synaptogenesis, cerebral metabolism and angiogenesis [23]. Various forms of exercise may be beneficial for neuroplasticity and applied especially for those suffering from cerebrovascular injuries and neurodegenerative disorders [24, 25].

## 3. Astrocytes Activation and Crosstalk Underly Exercise-Induced Neuroplasticity

Astrocytes, characterized by their intricate arborization have multiple receptors, transporters, and molecules; this feature enables them to sense neurons [26, 27]. Astrocytes release a variety of molecules at synaptic sites, including glutamate, d-serine, ATP, adenosine, lactate, and other soluble or contact factors that participate in the formation, stabilization, and remodeling of astrocyte-neuron or neuron-neuron crosstalk [28, 29]. Furthermore, astrocytes induce microglial activation and control their cellular functions. This astrocyte-microglia crosstalk is created through the release of a variety of molecules, including neurotransmitters, lipocalin proteins, inflammatory cytokines, and growth factors. It can be concluded that astrocyte activation and interactions in neural circuits after physical exercise can play a critical role in developing functional outcomes and neuroplasticity [30, 31].

### 3.1 Exercise Induces Astrocytes Activation

Studies have shown that physical exercise stimulates astrocytes proliferation, laying a cellular basis for mediating exercise-induced neurogenesis and improvements in cognitive function [32, 33]. Increasing evidences demonstrate that astrocytes can control the growth and differentiation of adult-born granule cells by releasing various trophic factors and gliotransmitters [34, 35]. In addition, they could supply energy, such as lactate, an essential component for neurogenesis [36]. The proliferation of astrocytes as well as their link to improved behavioral outcomes and neurogenesis may depend on releasing cytokines in GFAP-positive astrocytes, such as BDNF [37].

Astrocytes increase their overall arborization and distal-to-proximal span following continuous exercise [38, 39]. Additionally, astrocytes activated by physical exercise can affect the changes in neural activity by altering messenger RNA transcript expression. The expression of GFAP, thrombospondin 2 (Thbs2), leukemia inhibitory factor (Lif), and interleukin 6 (IL-6) are associated with astrocytes’ function during exercise. Physical exercise activates astrocytic gene expression by transcribing the different mRNA, which ultimately render the differences in astrocytic structure and function [40]. Exercise-induced astrocytic activation is thought to provide vascular protection against cerebral injuries, such as stroke, via vascular remodeling [41, 42]. Moreover, the astrocytic activation diminishes blood-brain barrier (BBB) dysfunction and promotes cerebral angiogenesis [43]. Overall, the aforementioned functions of astrocyte denote its role as a central component to exercise-induced neuroplasticity in many neurodegenerative and neurovascular disorders.

### 3.2 Exercise Induces Astrocyte-Neuron Crosstalk

A single astrocyte can be involved in hundreds of thousands of synapses [44]. This morphological feature is critical for the tight functional astrocyte-neuron crosstalk. Astrocytes regulate neurons by maintaining the crosstalk and delivering metabolic substrates to neurons [45]. In fact, physical exercise increases the level of astrocytes in the hippocampus, prefrontal cortex, striatum, and entorhinal cortex, and induces changes in their morphology [46]. These changes produce beneficial effects on neuronal activity and plasticity [39]. For instance, long-term exercise is found to induce the proliferation of astrocytes and improvements in learning and memory [47]. Increasing neural networks can promote brain recovery from cerebral injuries via neuroplasticity; alterations in astrocytes induced by physical exercise may be responsible for this effect [11]. Additionally, astrocytes express a variety of receptors for typical neurotransmitter molecules, including glutamate, acetylcholine, ATP, gamma-aminobutyric acid (GABA), norepinephrine, and release retrograde messengers such as endocannabinoids, which sense neural activity [29]. Neurotrophic factors and lactate are the primary source mediating astrocyte-neuron communication among a variety of molecules [29].

**Figure 1.**
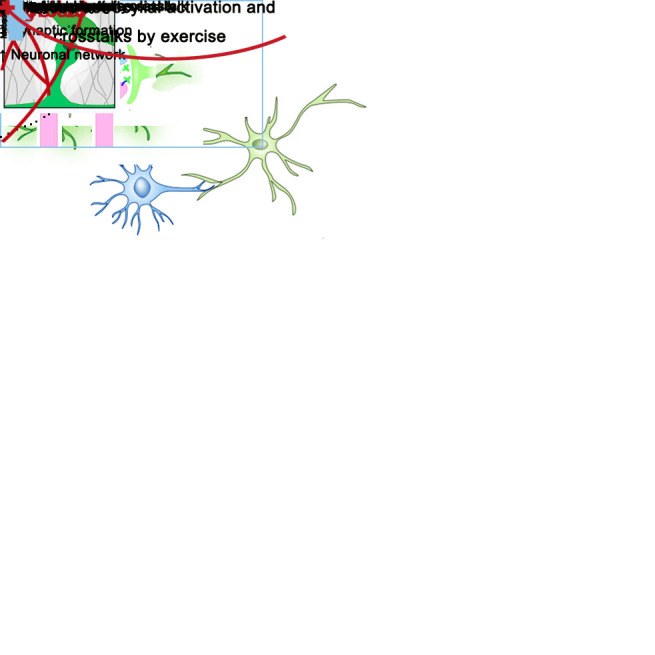
Exercise-induced astrocytes activation and crosstalk with neuron and microglia.

Exercise enhances astrocyte-neuron crosstalk by regulating neurotrophic factors [11], such as IGF-1 and BDNF. Astrocytes derived IGF-1 shows a protective effect on neurons through suppressing oxidative stress. In fact, the activation of IGF-1 by physical exercise initiates the involvement of astrocytes [48, 49]. In addition, physical exercise leads to an upregulation astrocyte expression of BDNF, thereby improving hippocampal neuroplasticity [50]. By acting on these neurotrophic factors derived from astrocyte, physical exercise mediates neural growth, proliferation, and survival, eventually enhancing neuroplasticity in numerous CNS disease [51].

Physical exercise also regulates energy metabolism of astrocytes to modify neuron activity, underlying its mechanism to the astrocyte-neuron crosstalk [52, 53]. Neurons generate energy mainly via oxidative phosphorylation, while astrocytes utilize glycolysis [54]. Lactate oxidation in mitochondria increases oxidative phosphorylation in neurons; lactate derived from astrocytes serves to promote the oxidative phosphorylation [55]. Lactate from astrocytes is delivered to neurons via the astrocyte-neuron lactate shuttle (ANLS) system, which has been reported to supply energy to the brain during physical exercise [56]. Moreover, physical exercise augments the effectiveness of the ANLS by upregulating astrocytic lactate transporter levels [57], suggesting a potential role of exercise in mediating astrocyte-neuron signal pathways. Figure 1 shows numerous ways that astrocytes and neurons interact and how they can impact neuroplasticity.

### 3.3 Exercise Induces Astrocyte-Microglia Crosstalk

Physical exercise has shown to affect numerous CNS pathologies by regulating astrocytes, microglia and glial activation [58-60]. There is growing evidence demonstrating that glial cells play a multifaceted role in ischemia and neurodegeneration; recent studies explain supportive effects of astrocytes and microglia on neural cell proliferation and survival [61]. Indeed, physical exercise results in increasing activation and crosstalk of microglia and astrocyte [62].

Emerging evidence indicates the bidirectional crosstalk between astrocytes and microglia occurs via their secreted molecules, including neurotransmitters, lipocalin proteins, inflammatory cytokines, and growth factors [63]. The molecular conversation between these cells plays a pivotal role in brain development, functions, and homeostasis [58]. A neurotransmitter, such as BDNF, derived from astrocyte, controls microglial activation, and reduces neurodegeneration [64]. Lipocalin proteins and inflammatory cytokines released from astrocyte regulate neuroplasticity via the relevant receptor-mediated signaling in microglia [65, 66]. Physical exercise mediates the crosstalk between astrocytes and microglia with regulatory effects on these molecules and cytokines to enhance neuroplasticity. Additionally, astrocytes and microglia are found to play beneficial roles in patient outcomes in certain neurological pathologies and even aid in brain development [58]. As Figure 1 illustrates, astrocyte-microglia interaction builds the foundation for the exercise-induced neuroplasticity. Nevertheless, this interplay is complex, thus, any detailed mechanism demands further investigations at this point.

### 3.4 Exercise Induces Microglia-Neuron Crosstalk

There are reciprocal and bidirectional crosstalk between microglia and neurons. Generally, microglial activation occurs is associated with dysfunctional neurons; physical exercise suppresses this detrimental effect by regulating microglia, neurons and their crosstalk [67, 68]. In addition to their role as an immune sentinel in the brain, microglia express a large variety of neurotransmitter receptors which in?uences the key microglial functions, such as cytokines production, cellular motility and phagocytosis [69]. Neurons also express receptors that are activated by the molecules from microglia [63]. Physical exercise triggers communications between microglia and neurons through microglial activation, neural regeneration and cytokine secretion. For instance, CD200-CD200R, ATP, and CX3CL1-CX3CL1R interaction pathways are shown to be enhanced by exercise [70, 71].

Microglial activation occurs with dysfunctional neurons. The ability of microglia to engulf dysfunctional neural synapses is impaired in a pathological state [72]. Physical exercise could ameliorate this abnormality by activating neurons whose axons provide synaptic contacts with pyramidal cell dendrites [73]. Current studies indicate the microglia-neuron crosstalk is induced by exercise and plays as an important contributing factor to neuroplasticity as illustrated in Figure 1.

## 4. Molecular Pathways Underlying Exercise-Induced Astrocyte Crosstalk

### 4.1 Signals Pathway Underlying Neurotransmitter of Astrocyte-Neuron Crosstalk

**Figure 2.**
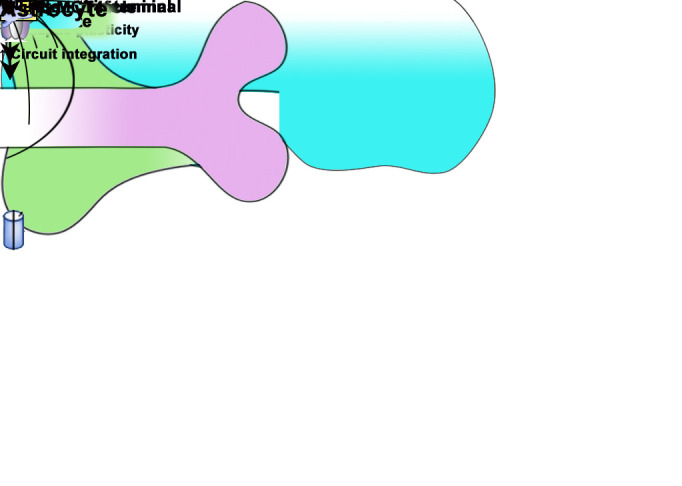
Signal pathway underlying astrocyte-neuron crosstalk

Astrocytes are able to enhance neuroplasticity by releasing mediators which directly modify neuron function which are illustrated in Figure 2 [74, 75]. This ability appears to play an important role in neuroplasticity [76]. Glutamate, an essential excitatory neurotransmitter, activates stimulatory presynaptic N-methyl-d-aspartate receptors (NMDAR) via astrocyte activation [8, 77]. Astrocytes are speculated to release glutamate via Ca^2+^-dependent vesicular exocytosis, regulating synaptic transmission, activities of neurons, and neuroplasticity [75]. Physical exercise could mediate astrocyte activation and astrocyte-neuron crosstalk by regulating glutamate release [52, 78] (Fig. 2). It has been found that physical exercise improves neuroplasticity by increasing the density of GFAP positive astrocytes, glutamine secreted from astrocyte [79], and NMDAR expression in neurons [80]; these changes indicate potential role of astrocyte on regulating neurons and there crosstalk.

D-serine, a neurotransmitter released by the astrocytes, has shown to play a role in the classical NMDAR-dependent long-term potentiation (LTP) [81, 82] (Fig. 2). D-serine takes an important part in forms of neuroplasticity by integrating adult-born granule neurons into the hippocampal circuitry, which affects local neural circuit performance in memory processes and mood control [34, 83]. Exercise induces the release of D-Serine from astrocytes, and this may explain the underlying astrocyte-neuron interactions [84].

*Lactate*. Lactate from astrocytes has shown to play a vital role in LTP at neural synaptic sites [85, 86] (Fig. 2). In an active metabolic state, glycogen from astrocytes are converted to lactate and delivered to neurons [85, 87]. Lactate affects neurons in many ways [88, 89]. It activates NMDAR and stimulates neuronal molecules, such as the cell-surface lactate receptor GPR8139-41 [90]. Physical exercise has been reported to increase the astrocyte-neuron metabolic shuttle by upregulating the astrocytic lactate transporter levels to induce neuroplasticity [57]. Exercise increased glutamate concentration and decreased lactate levels hence demonstrating lactate transport from astrocytes to neurons; these studies underly the increasing astrocyte-neuron crosstalk induced by exercise.

*Tumor Necrosis Factor-α (TNFα)*. Astrocytes modulate synapses and neuroplasticity with immune mediators that are produced in physiological and pathological inflammatory reactions [91, 92]. For instance, TNFα induces synaptic remodeling by incorporating AMPA receptor subunits in excitatory synapses [93] and influences the neuron activity by regulating the release of glutamate from astrocytes [94] (Fig. 2). Studies indicate physical exercise improves neural function and reduces neuron damage by regulating the activation of astrocytes and release of TNF-α [95]. Therefore, TNFα derived from astrocyte have potency to mediate neurons and an inflammatory response to be a potential source altering neuroplasticity.

**Figure 3.**
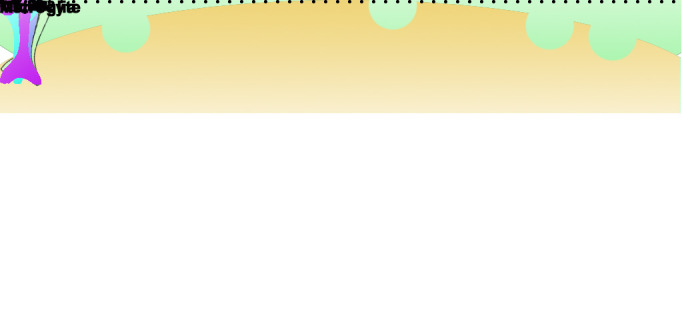
Signaling pathways underlying astrocyte-microglia crosstalk.

### 4.2 Signals Pathway Underlying Astrocyte-Microglia Crosstalk

Microglia, the immune cell of the CNS, exert neurotoxic and neuroprotective effects and regulate astroglial function and neuroplasticity [96]. Microglia affect neurotransmissions and appear to be necessary for neuronal reorganization in the adult brain [63]. Microglia build the neuronal network by inducing synapses with astrocytes [58]. Although the interplay between astrocytes and microglia remains unclear, several molecules responsible for the astrocyte-microglia crosstalk have been elucidated in Figure 3.

*Neurotransmitters*. Increasing numbers of studies indicate that targeting the microglia-astrocyte crosstalk has a therapeutic potential [58]. Numerous membrane receptors enable microglia to communicate with astrocytes while mediating neuronal activity and synaptic transmission [97]. For instance, microglia release ATP that could induce astrocytes to release glutamate, indicating the astrocyte-mediated regulation of excitatory neurotransmission [98]. Physical exercise is reported to increase the proportion of anti-inflammatory M2 microglia with induced ATP synthesis [43]. These findings point out a potential role of exercise on astrocyte by regulating microglia release of ATP. Additionally, glial-cell line-derived neurotrophic factor (GDNF), cerebral dopamine neurotrophic factor (CDNF), and BDNF are some of the important astrocyte molecules that are involved in the modulation of the microglial activation [58, 99]. Physical exercise is reported to save neurons as well as induce microglia activation by increasing the astrocyte-derived BDNF and GDNF levels [50]; these denote the potential neuroplastic effect of physical exercise with the neurotrophic factors from the microglia-astrocyte interaction (Fig. 3).

*Lipocalin Proteins*. Studies have shown that astrocyte-derived orosomucoid-2 (ORM2) and lipocalin-2 (LCN2) induce the microglial activation [66, 100]. Mainly secreted by astrocytes, ORM2 binds to the microglial C-C chemokine receptor type 5 (CCR5) and inhibits the CXCL4-CCR5 interaction, which is critical for the microglial activation [101]. LCN2 is found in astrocytes whereas the LCN2 receptors (LCN2R) are mainly expressed on microglia [102]. The distribution of LCN2 and LCN2R suggests that the astrocyte-derived LCN2 could act on microglia [100]. In fact, a series of experiments show that astrocytes in the hippocampus interact with microglia by secreting LCN2 in the rodent brains [66]. According to previous studies, physical exercise upregulates ORM level [103, 104] and LCN2 [105, 106]. Improving neuroplasticity and behaviors are found in lipocalin 2-null mice after exercise [107]; these findings note a potential effect of exercise on neuroplasticity by ORM2 or LCN2 from the astrocyte-microglia interaction (Fig. 3).

*Inflammatory Cytokine*. Interleukin-33 (IL-33) is a vital IL-1 family member as a cellular alarmin especially after tissue damage, such as spinal cord injuries, stroke, and Alzheimer’s disease (AD) [108-110]. In addition to its roles in inflammation, IL-33 promotes brain tissue development and remodeling [111, 112]. In the brain, astrocytes are the primary source of IL-33 and microglia mainly express ST2 receptors [113, 114]. Studies demonstrate that astrocyte-derived IL-33 promotes microglial synapse engulfment and neural circuit development via the ST2 receptor-mediated signaling in microglia [113]. The increasing levels of IL-33 and ST2 are also found after physical exercise in normal people or those with ischemic heart disease [115]; these findings suggest that exercise-induced IL-33/ST2 maybe associated with astrocyte-microglia crosstalk (Fig. 3).

CXCL12 and CXCR4 are found to be expressed in astrocytes and microglia respectively, implying the CXCL12/CXCR4 axis may be involved in the astrocyte-microglia crosstalk [116]. Studies indicate that crosstalk between astrocytic CXCL12 and microglial CXCR4 are related to the development of neuropathic disease. Furthermore, CXCL12 and CXCR4 are upregulated in rats that have undergone constrained exercise on a treadmill [117]. Physical exercise is found to regulate the neural stem cell proliferation and migration via the CXCL12-CXCR4 pathway in rats after ischemic stroke [118]. This evidence suggests a potential role of physical exercise on the astrocyte-microglia crosstalk through the CXCL12-CXCR4 signaling (Fig. 3).

Microglia regulate the astrocyte activation by releasing IL-1 whose receptors are mainly expressed on astrocytes [119]. Microglia activates astrocytes by secreting IL-1 which promotes neuronal survival, outgrowth and neuroplasticity in various human neurodegenerative diseases [97]. Physical exercise has a strong effect on the immune system and induces the production of IL-1 [120] and IL-1 receptors [121]. These studies denote that physical exercise may modify the astrocyte-microglia crosstalk through IL-1. Additionally, exercise-induced inflammatory molecules mediating the microglia-astrocyte crosstalk also include C3, MCP-1, TNFα and C1q [122-125] (Fig. 3).

**Figure 4.**
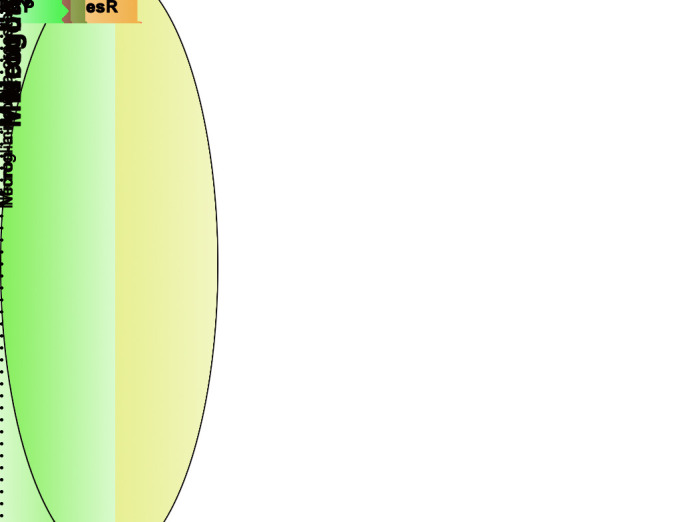
Signals underlying crosstalk between microglia and neuron.

### 4.3 Signals Underlying Exercise-Induced Crosstalk Between Microglia and Neuron

Microglia can directly influence neuronal activity and neuroplasticity through many signals [63, 126] (Figure 4). Interactions between microglia and neurons play an important role in many neurological disorders with altered neural network excitability [127]. Advanced understanding in the neurobiology field has revealed active roles of microglia in modulating neuron activities, glial function, the crosstalk between neurons and microglia [70]. Anti-inflammatory microglia are found to enhance neuronal growth, suggesting microglia’s concomitant growth-promoting properties [63].

CX3CL1 is one of the critical chemokines released from neurons and binds to microglial receptors, CX3CR1 [128, 129]. Studies have indicated that decreased microglia-neuron communication caused by reduction of CX3CR1/CX3CL1 signaling, is critical for the loss of the homeostatic microglial function [63]. Physical exercise is believed to improve the cognitive function and neuroplasticity through the CX3CL1-CX3CR1 signal mediated by microglia [130]. Studies address that physical exercise could modulate stress-evoked neuronal-microglial responses by altering the CX3CL1-CX3CR1 axis, which results in activation and proliferation of microglia and neuroplasticity [70]. These findings suggest that exercise-induced microglial-neuron communications somehow depend on the CX3CL1-CX3CR1 axis (Fig. 4).

Microglia modulate neuron activities and refine neural circuits through the complement system [131]. Complement and microglia are found to mediate early loss of synapsis in neurodegenerative disorders [132]; inhibiting C3, C1q, or the microglial complement receptor, CR3 decreases the extend of early synaptic loss and the number of microglia [133]. Microglia-mediated neurotoxic effects in neurodegenerative diseases could be explained by astrocytes activation induced by cytokines and C1q [97]; this mechanism implies that microglia may indirectly affect neurons with reactive astrocytes. Long-term physical exercise has been reported to prevent pathological neurovascular changes by reducing C1q^+^ microglia and increasing neuroplasticity [134]; these findings indicate that physical exercise may induce microglial-neuron communication through complement molecules (Fig. 4).

Neuron-microglia crosstalk also occurs through interaction between a neuronal glycoprotein, CD200, and a microglial receptor, CD200R, leading to a formation of CD200-CD200R complex [135, 136]. Interaction of neuronal CD200 and microglial CD200R are associated with neurodegenerative changes. Alteration in the activation of CD200 and microglia in those with depression have been observed after treatment with physical exercise [130]. Studies have established that exercise prevents dopaminergic neuronal loss by suppressing brain inflammation and microglial activation and increasing expressions of CD200 and CD200 receptor [71]. These findings suggest physical exercise could mediate the neuron-microglia crosstalk via the CD200-CD200R signaling (Fig. 4). Additionally, more pathways mediated by exercise are demonstrated to address the communications between neurons and microglia [137-148].

## 5. Exercise-Induced Astrocyte Activation/Crosstalk in Nervous System Disease

Physical exercise improves brain functions by regulating glial activation in numerous CNS diseases, including AD, Parkinson’s Diseases and ischemic stroke [60, 149, 150]. Physical exercise could activate cellular and molecular pathways contributing to neuroplasticity [4, 151]. Glial cells play a multifaceted and complex role in CNS diseases with their supportive effects on cell proliferation and brain plasticity [61]. Neuroplasticity is found to be enhanced in the absence of microglia T cell interaction and microglia activation after exercise [62]. Physical exercise inhibits neuroinflammation and microglial activation and modulates dopaminergic damages [60, 149].

## 6. Conclusion

This article highlights the neuroplastic effects of exercise on astrocytes and their interplays with microglia and neurons. Physical exercise promotes the astrocytic proliferation, astrocytic transporter levels, and interaction between glial cells and neurons. These effects help to increase the number of synapses, neural structures, and pre- and postsynaptic receptor localization. Future studies are needed for further clarification on the complex interactions between exercise, glial cell functions, and other key molecular mediators.
